# Lobular Capillary Hemangioma Masquerading as Pyogenic Granuloma of Anterior Mandible: A Case Report

**DOI:** 10.7759/cureus.42157

**Published:** 2023-07-19

**Authors:** C V Srinivedha, Dr Saurabh Simre, Abiskar Basnet, Sameer Pandey, Ashi Chug

**Affiliations:** 1 Oral and Maxillofacial Surgery, All India Institute of Medical Sciences Rishikesh, Rishikesh, IND; 2 Craniomaxillofacial Surgery, All India Institute of Medical Sciences Rishikesh, Rishikesh, IND

**Keywords:** lch, recurrent, pyogenic granuloma, gingiva, lobular capillary hemangioma

## Abstract

Pyogenic granuloma (PG) is a reactive connective tissue disorder with female predilection, which exhibits a tumor-like mass with occasional bleeding and superficial ulceration. It most commonly occurs in the maxillary gingiva followed by the mandibular gingiva. It can also occur in extra gingival sites like buccal mucosa, labial mucosa, and palate. There are two histopathological types of PG, namely, a lobular capillary hemangioma (LCH) variant and a non-LCH variant.

The various management methods include surgical resection or laser excision along with deep curettage, and there are various nonsurgical methods like local steroid injection, topical administration of various drugs, and sclerotherapy. During the surgical excision, there is a risk of bleeding, and the surgeon should be equipped for the same. The PG (both LCH and non-LCH variant) has an increased chance of recurrence because of which complete excision is mandatory along with the removal of the local irritants.

In this case report, a 28-year-old female patient reported recurrent painless swelling in the lower front gums for the past nine months.

The surgical excision was done in-toto along with the removal of local irritants (calculus). The swelling was sent for histopathological examination. The patient was kept on regular follow-ups.

The patient was followed up continuously for nine months. The swelling did not recur after the excision.

Hence, it was concluded that complete excision and removal of local irritants are extremely crucial to prevent a recurrence.

## Introduction

Pyogenic granuloma (PG) is a reactive benign lesion of the mucosa. The name PG is a misnomer because there is no evident pus in the lesion. The lesion was described by Hartzell in 1904. This is the most commonly occurring growth in the oral cavity, with various predisposing factors such as trauma and local irritation [1]. The same entity was proposed as “hemangiomatous granuloma” by Angelopoulos, which precisely gives the histopathologic picture (both inflammatory component and hemangioma) [1]. Ver Berne et al. suggested an alternative term "granuloma telangiectacticum" for PG due to the presence of numerous blood vessels [2].

In 2003, Toida et al. described the two variants of the lesion, namely, the lobular capillary hemangioma (LCH), also called epulis gravidarum, and the non-LCH [3]. However, the WHO classification of head and neck tumors (2017) has described both entities as synonyms and classified them under hemangiomas, while in 2018, the International Society for the Study of Vascular Anomalies (ISSVA), has described both entities as benign tumors [2]. Although both LCH and non-LCH are considered subtypes of PG, Ver Berne et al. described the clinical, pathological, and histopathological differences between PG and LCH [2]. According to them, the LCH is known to arise as a result of vascular malformation, occurs spontaneously, and is of the non-LCH type, i.e., PG is due to the hormonal changes that occur as a result of pregnancy or puberty. PG has chaotically arranged densely packed capillaries, while LCH has uniformly arranged organized lobules of capillaries [2].

Mahajan et al. reported that this lesion has increased female predilection with more prevalence in young adults and teenagers [4]. 

This lesion (PG/LCH) may develop rapidly and reach full size. The etiology of this entity is still an enigma. The lesion is thought to arise from local factors such as recurrent trauma, calculus, and also hormonal changes during pregnancy, and the reproductive age group could predispose to the above lesion. This lesion is unique in a way that it can recur many times even after complete excision and can bleed intraoperatively. Lobular capillary hemangioma could be considered a separate entity from PG and would recur frequently if not managed properly in the first visit.

The present case report describes a recurrent LCH variant of PG in a 28-year-old female patient along with its successful management.

## Case presentation

A 28-year-old female reported to the outpatient department with a chief complaint of recurrent swelling in her gums in the lower front teeth region for the past nine months. The swelling was insidious in onset and gradually increased to the present size with no pain or pus discharge or any bleeding points. The patient had reported similar swelling twice before, which was excised under local anesthesia by a private practitioner. Then the swelling recurred within a span of two months. No previous documents were provided by the patient. The patient opted for excision of the swelling due to esthetic issues.

**Figure 1:**
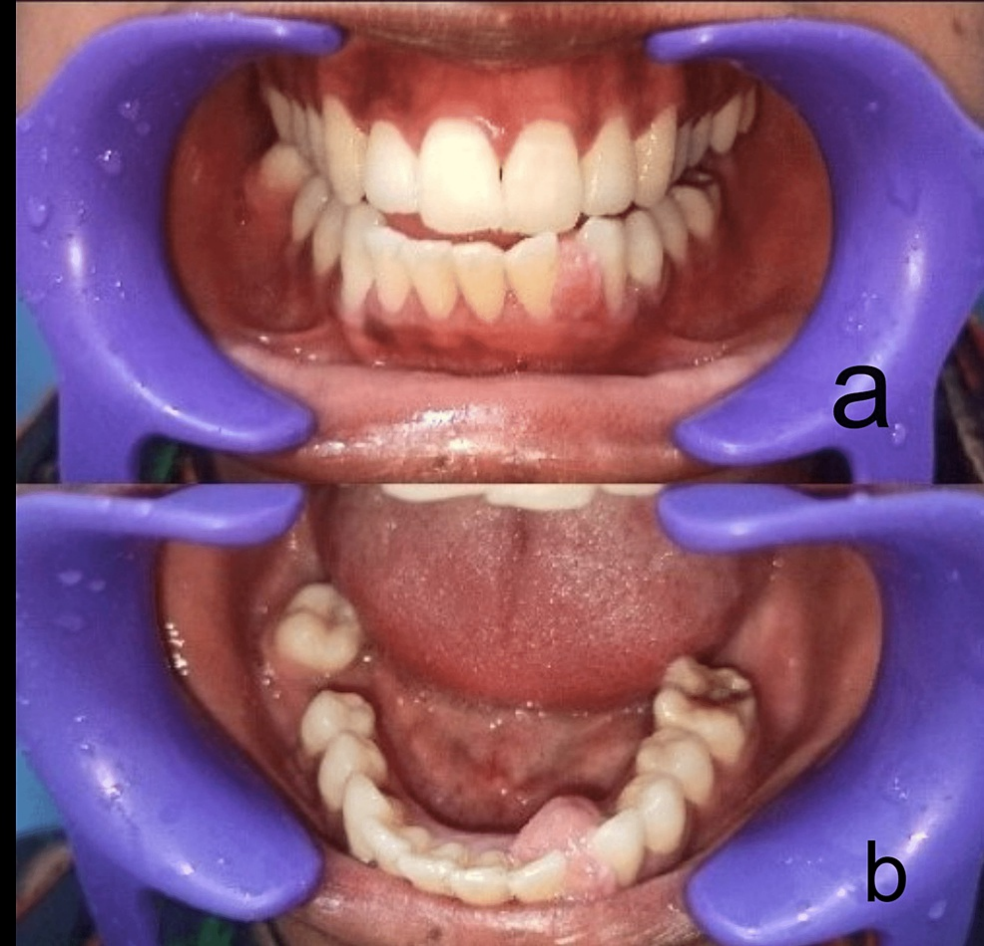
Preoperative picture of the patient (a) Swelling observed in the buccal gingiva between 32 and 33 and (b) swelling observed in the lingual gingiva between 32 and 33.

On inspection, there was a single, ovoid swelling in the mandibular anterior region between 32 and 33 with the same color as adjacent mucosa, approximately 2 cm x 1.5 cm in size, with well-defined margins and well-defined border, with no active pus discharge. On palpation, the swelling was soft in consistency, non-tender, and had no fixity to underlying tissues. There was no pus discharge, fluid discharge, or bleeding on palpation. The swelling was 2 cm x 1.5 cm x 0.5 cm in size on palpation. On hard tissue examination, the patient had calculus in all the mandibular anterior regions. Preoperative photographs are presented in Figure 1.

**Figure 2:**
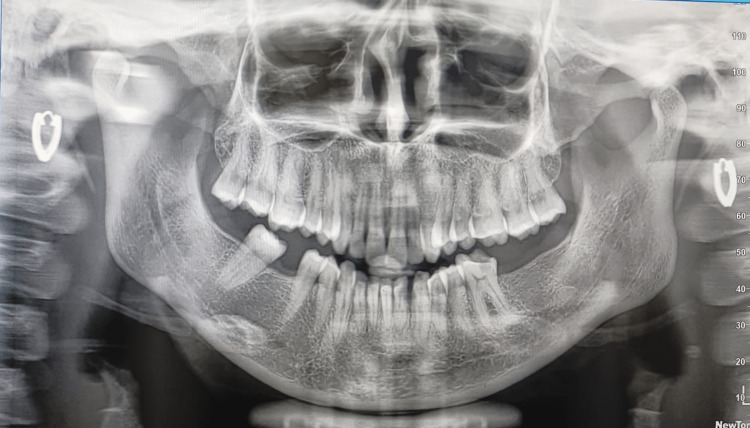
Panoramic radiograph of the patient The panoramic radiograph shows complete permanent dentition with no bone loss.

Figure 2 depicts a panoramic radiograph that revealed no local radiolucency and was unremarkable. Supragingival scaling was done one week prior to the swelling excision to remove the calculus and local irritants. The swelling was excised in-toto, from both the buccal and lingual sides through an intra-oral crevicular approach under local anesthesia, and deep curettage was done with respect to 32 to 33 at the same visit to remove the local irritants. Bleeding was anticipated during the surgery, but the lesion was excised in-toto uneventfully, and suturing was done with 3-0 Vicryl. Post-surgery, the patient was instructed to maintain oral hygiene (brushing twice daily with a soft toothbrush and toothpaste, warm saline rinses five to six times per day, and chlorhexidine rinses two times per day for two weeks).

**Figure 3:**
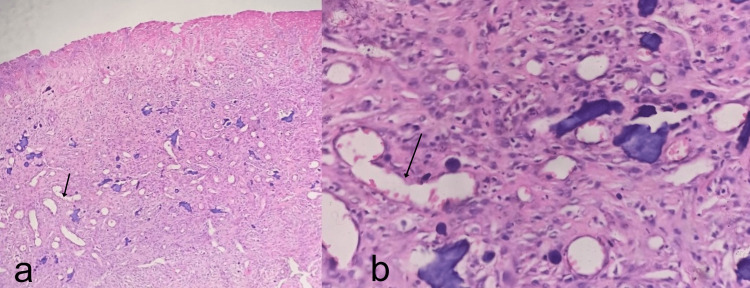
Histopathological pictures (a) Low-power view (20X) shows the lesion comprised of dilated congested thin-walled vessels and (b) high-power view showing ectatic blood vessels with a single lining of endothelial cells and lumen filled with red blood cells.

The histopathological examination revealed LCH, which showed a lesion comprised of dilated congested thin-walled vessels in low-power view (20X) and ectatic blood vessels in high-power view with a single lining of endothelial cells and lumen filled with red blood cells. The histopathological picture is shown in Figure 3.

**Figure 4:**
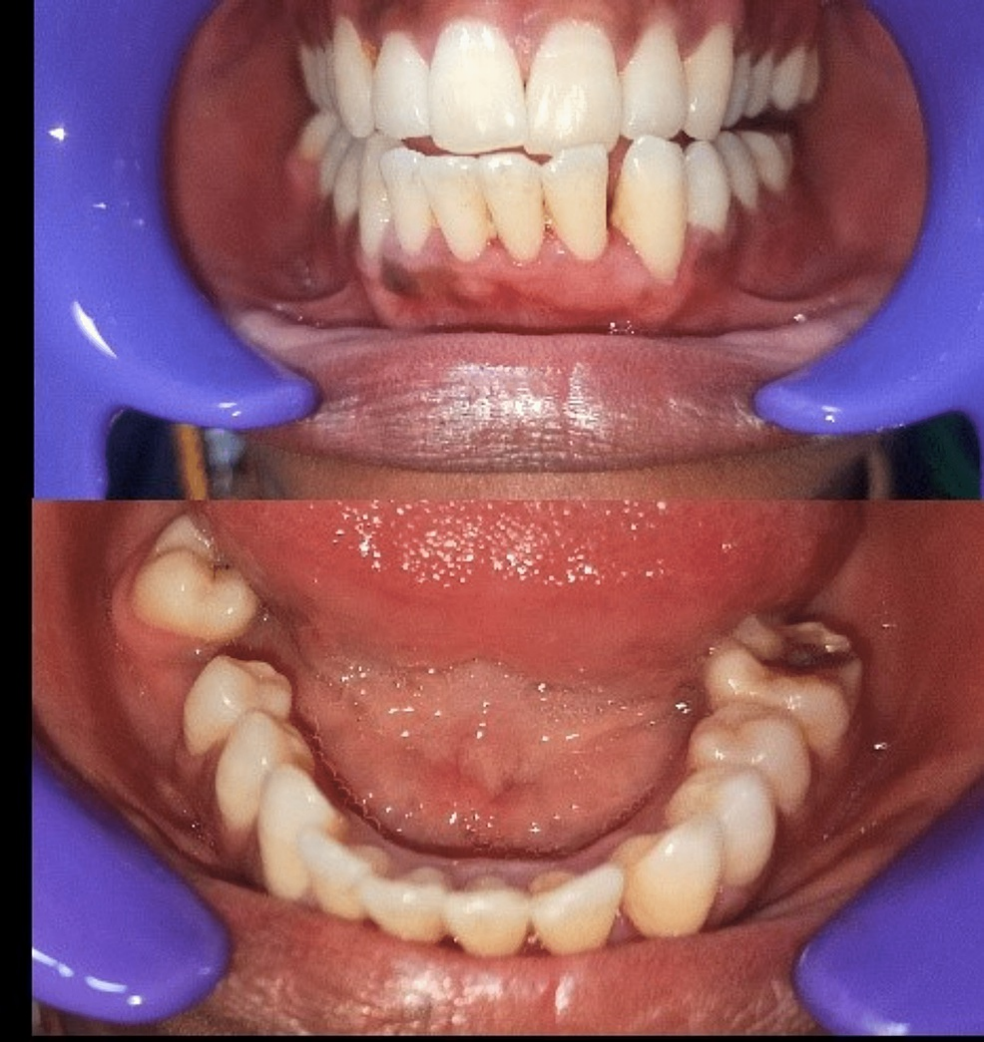
Postoperative pictures This image shows the complete resolution of the swelling with no recurrence for nine months.

The patient was followed up for nine months, and no signs of recurrence were evident. Postoperative pictures are shown in Figure 4.

## Discussion

PG has slight female predilection, and it frequently occurs in the second and third decades. Literature reports pinkish or purplish colored nodular or globular mass with occasional superficial ulceration. This lesion can also occur in pregnancy and is named as a pregnancy tumor, which mostly resolves spontaneously post-childbirth [5]. Hemangioma is a vascular proliferation of blood vessels. It commonly occurs in children less than one year of age. They can be congenital or can be acquired in the early stages of life. PG has two types, one of which is non-LCH. The two variants have minute differences in etiopathogenesis, clinical features, and histopathological features [2].

LCH is also a fast-growing lesion, with a mean diameter of 0.8 cm, making it relatively smaller [6,7]. In the oral cavity, the occurrence is most common in the maxillary gingiva. It can also occur in other sites like labial and buccal mucosa, tongue, mandibular gingiva, and palate [6]. Bleeding is frequently encountered during surgical excision of the same [5]. The characteristic histologic feature of LCH is proliferating blood vessels, which are arranged in lobular aggregates. The foci of focal maturation are seen in the non-LCH variant of PG.

While the pathogenesis of PG root tracks the effect of estrogen, with predisposing factors such as pregnancy and puberty, LCH has no known stimulus to stimulate the bone marrow stem cells to get differentiated into ovoid cells, endothelial cells, and pericytes. Ovoid cells interact with the pericyte, and it secretes tyrosine receptors to induce angiogenesis and neovascularization. As a result, there occurs a proliferation of endothelial cells, smooth muscle cells, and ovoid cells, thus giving rise to the lesion [2].

The various differential diagnoses include peripheral giant cell granuloma, which shows multinucleated giant cells upon histological examination. Another differential diagnosis is Kaposi’s sarcoma, which has pathognomic features such as the proliferation of dysplastic spindle cells, extravasated erythrocytes, vascular clefts, and intracellular hyaline globules.

Surgical excision along with the removal of local irritants is the treatment of choice as per the literature. Laser-assisted excision (Nd:YAG laser) is also a viable option for the excision of PG [8]. The lesions not amenable to surgery could be managed by nonsurgical modalities like local steroid injection with triamcinolone acetonide at a dose of 0.1 ml of 10 mg/ml [9]. Topical timolol application or injection of ethanol can also be used due to the vasoconstrictive action of beta blockers, which is believed to inhibit the angiogenic factors owing to the regression of the lesion. The use of sclerosing agents like sodium tetradecyl sulfate has also been documented in the literature [10]. Alitretinoin gel 0.1%, which is commonly used for Kaposi’s sarcoma, is also promising in the management of PG or LCH due to the histologic similarities of both lesions [7]. Low-dose brachytherapy is being tested in the literature. Follicular stimulating hormone antagonists can prevent recurrence post-excision [6].

The recurrence rate is 8%-15% with females having an increased recurrence rate [6,7]. Generally, lesions in the gingiva recur more frequently than the other mucosal sites. The main reason for recurrence is insufficient removal of the lesion or failure in the removal of the local irritants. Various causes responsible for recurrence include angiopoietin 1,2, ephrin b2, viral oncogenes, hormonal influence, microscopic arteriovenous malformation, and gene depression in fibroblasts [10]. In the present case, the recurrence might be due to the non-removal of local irritants, and the lesion did not recur when the local irritants such as calculus were removed. For the management of recurrent lesions, surgical excision is described as a treatment of choice [10]. However, various other nonsurgical modalities have been quoted in the literature such as the injection of absolute ethanol, corticosteroids, and sodium tetradecyl sulfate sclerotherapy [10].

## Conclusions

As proposed by ISSVA, the LCH variant of PG could be considered a vascular tumor and a separate entity from PG. Complete excision of the tumor should be done and all the local irritants should be removed in order to prevent recurrence. During the surgery, precautions should be made to prevent bleeding, and the surgical team should be ready to tackle the emergency.
